# Regulation of Retinoid-Mediated Signaling Involved in Skin Homeostasis by RAR and RXR Agonists/Antagonists in Mouse Skin

**DOI:** 10.1371/journal.pone.0062643

**Published:** 2013-04-24

**Authors:** Janine Gericke, Jan Ittensohn, Johanna Mihály, Susana Álvarez, Rosana Álvarez, Dániel Töröcsik, Ángel R. de Lera, Ralph Rühl

**Affiliations:** 1 Department of Biochemistry and Molecular Biology, University of Debrecen, Debrecen, Hungary; 2 Paprika Bioanalytics BT, Debrecen, Hungary; 3 Departamento de Química Orgánica, Universidade de Vigo, Vigo, Spain; 4 Department of Dermatology, University of Debrecen, Debrecen, Hungary; 5 Apoptosis and Genomics Research Group of the Hungarian Academy of Science, Debrecen, Hungary; University Hospital Hamburg-Eppendorf, Germany

## Abstract

Endogenous retinoids like all-*trans* retinoic acid (ATRA) play important roles in skin homeostasis and skin-based immune responses. Moreover, retinoid signaling was found to be dysregulated in various skin diseases. The present study used topical application of selective agonists and antagonists for retinoic acid receptors (RARs) α and γ and retinoid-X receptors (RXRs) for two weeks on mouse skin in order to determine the role of retinoid receptor subtypes in the gene regulation in skin. We observed pronounced epidermal hyperproliferation upon application of ATRA and synthetic agonists for RARγ and RXR. ATRA and the RARγ agonist further increased retinoid target gene expression (Rbp1, Crabp2, Krt4, Cyp26a1, Cyp26b1) and the chemokines Ccl17 and Ccl22. In contrast, a RARα agonist strongly decreased the expression of ATRA-synthesis enzymes, of retinoid target genes, markers of skin homeostasis, and various cytokines in the skin, thereby markedly resembling the expression profile induced by RXR and RAR antagonists. Our results indicate that RARα and RARγ subtypes possess different roles in the skin and may be of relevance for the auto-regulation of endogenous retinoid signaling in skin. We suggest that dysregulated retinoid signaling in the skin mediated by RXR, RARα and/or RARγ may promote skin-based inflammation and dysregulation of skin barrier properties.

## Introduction

The nuclear hormone receptors retinoic acid receptors (RAR) α, β, and γ and retinoid X receptors (RXR) α, β, and γ are ligand-dependent transcription factors that can be activated by retinoids. RAR-RXR heterodimers regulate the expression of multiple genes in skin and various other tissues [1], while their transcriptional activity is dependent on the RAR-activating ligand [2]–[4]. The most abundant RAR and RXR subtypes in skin are RXRα and RARγ, followed by lower quantities of RARα [5]. Since retinoid receptors exhibit tissue and cell type-specific distribution patterns, functional specificity of each subtype is suggested [6]–[12]. Moreover, RAR and RXR subtypes differ in ligand specificity and/or affinity [9], [11]–[14], therefore, it can be assumed that their contribution to gene expression patterns in skin differs, depending on quantitative receptor distribution, on the nature and level of co-regulators, as well as on available retinoid receptor-selective agonists and antagonists.

RAR-RXR-mediated signaling pathways induced by retinoids are essentially involved in immune-modulatory events [15]–[17], and skin physiology [18] through their role in the regulation of several aspects of skin cell proliferation, differentiation, apoptosis, and epidermal barrier function [19], [20]. Retinoid metabolism and concentrations in skin are tightly regulated ensuring sufficient levels of the endogenous pan-RAR activator all-*trans* retinoic acid (ATRA) [2], [21], [22]. However, alterations in retinoid metabolism, signaling and concentrations have been observed in various dermatoses, such as psoriasis [23], ichthyosis [24], and recently in a study by our group in atopic dermatitis [25]. Altered retinoid-mediated signaling in skin of these patients may also be a result of activation or antagonism of specific retinoid receptor subtypes under disease conditions.

In order to dissect retinoid-mediated signaling in skin, mice were treated topically for two weeks with selective RAR and RXR agonists or antagonists. Our aim was to determine the effect of RAR subtype-selective and RXR activation or antagonism on the expression of genes involved in retinoid metabolism and signaling, as well as epidermal barrier homeostasis and skin-based immune regulation. The outcome of the present study will help to identify pathways and genes that are selectively regulated by RARα, RARγ, or RXR in the skin of mice. This might allow for conclusions regarding the involvement of subtype-specific retinoid receptor-mediated signaling in various skin diseases and may suggest alternative therapeutic strategies.

## Materials and Methods

### Retinoid Receptor-specific Agonists and Antagonists

ATRA was a gift from BASF (Ludwigshafen, D) and the synthetic RXR activator LG268 was kindly provided by Ligand Pharmaceuticals (San Diego, CA). Synthetic agonists selective for RARα (BMS753) and RARγ (BMS189961) were prepared in our laboratories as described in the original patents [26], [27] with the yields indicated as supporting information (Figure S1 and S2). The RARα-specific antagonist (BMS614) was made following the patented procedure developed at BMS [28], [29] as detailed in the supporting information section (Figure S3). The RARγ-selective antagonist (UVI2041) was prepared by the condensation of the ester **15** derived from chalcone **14** [30] with hydroxylamine [31], [32] followed by hydrolysis as described in supplements (Figure S4). The RAR pan-antagonist/inverse agonist (BMS493) and the RXR pan-antagonist (UVI3003) were synthesized according to reported procedures [33], [34]. The purity of the synthesized compounds was determined to be greater than 95% by HPLC after crystallization. We have confirmed that these retinoids are stable when stored as solids or in solution at −78°C, and during the time frame of biological experiments.

### Sensitization of Mice

8–12 weeks old female C57BL6 mice were obtained from and housed within the animal facility of the University of Debrecen, Hungary. Animals were maintained in single cages on standard animal chow and water *ad libitum*. All experimental procedures were approved by the Committee of Animal Research of the University of Debrecen, Hungary (Approval number: 25/2006 DEMÁB).

Mice were anesthetized and subsequently shaved on dorsal skin sites using an electric razor. Retinoid receptor-specific agonists and antagonists were applied topically each other day in 25 µl acetone (vehicle/control; Merck, Darmstadt, D) per treatment for two weeks. According to previous studies by other groups [2], [35] agonists and antagonist were applied in the following concentrations: ATRA, 40 nmol; LG268, 100 nmol; BMS753, 40 nmol; BMS189961, 40 nmol; BMS614, 100 nmol; UVI2041, 100 nmol; BMS493, 100 nmol; UVI3003, 100 nmol. On day 14, four hours after the last treatment, mice were sacrificed, sera and full thickness skin biopsies were collected, skin specimen were shock frozen in liquid nitrogen and all samples were kept at −80°C until analyses. Skin samples were obtained from equal body sites by means of the same procedure for each mouse in order to control for variability among specimen. Samples were visibly controlled to ensure no excessive adipose tissue remained, though some contamination with remaining adipose tissue cannot be excluded.

### RNA Preparation and Reverse Transcription

Total RNA was isolated from frozen full thickness skin biopsies using Tri® reagent (Molecular Research Center Inc., Cincinnati, OH) following the manufacturer’s instructions. Concentration and purity of RNA samples were determined with NanoDrop spectrophotometer (Thermo Scientific, Budapest, H). 750 ng of total RNA were reverse transcribed into cDNA in a 30 µL reaction mix using the High Capacity cDNA Reverse Transcription Kit (Life Technologies, Budapest, H) according to the manufacturer’s protocol.

### Analysis of mRNA Expression

mRNA expression in total skin was determined by means of quantitative real time-PCR (qRT-PCR) on an ABI Prism 7900. Measurements were performed in triplicate using pre-designed TaqMan® Gene Expression Assays and reagents (Applied Biosystems Applera Hungary, Budapest, H). Relative quantification of mRNA expression was achieved using the comparative C_T_ method and values were normalized to cyclophilin A mRNA. Additionally, Gapdh gene expression was determined to confirm that house keeping gene expression was not affected by the various treatment regimens (not shown). Gene expression values below detection limit were assumed to be zero for the purpose of statistical analysis.

### Histological Analysis

Skin biopsies were taken from similar dorsal body sites and kept at −80°C until analysis. Frozen specimens were sectioned (4 µm) and stained with hematoxylin and eosin (H&E).

### Determination of All*-trans* retinoic Acid Levels in Skin

Concentrations of ATRA were determined in mouse skin samples by our high performance liquid chromatography mass spectrometry - mass spectrometry (HPLC MS-MS) method as described previously [36]. In summary, 100 mg of skin biopsy (if samples were under 100 mg, water was added up to the used standard weight: 100 mg) were diluted with a threefold volume of isopropanol, tissues were minced by scissors, vortexed for 10 seconds, put in an ultra sonic bath for 5 minutes, shaken for 6 minutes and centrifuged at 13000 rpm in a Heraeus BIOFUGE Fresco at 4°C. After centrifugation, the supernatants were dried in an Eppendorf concentrator 5301 (Eppendorf, Germany) at 30°C. The dried extracts were resuspended with 60 µl of methanol, vortexed, shaken, diluted with 40 µl of 60 mM aqueous ammonium acetate solution and transferred into the autosampler for subsequent analysis.

### Statistical Analysis

Data are indicated as mean ± SEM. Statistical analysis of qRT-PCR data was performed using one-way ANOVA followed by Dunett’s post-test. Significance of HPLC MS-MS results was determined using Student’s *t*-test. Differences were considered significant at *p*<0.05.

## Results

### ATRA and a Synthetic RARγ agonist Induce Epidermal Hyperproliferation

**Figure 1:**
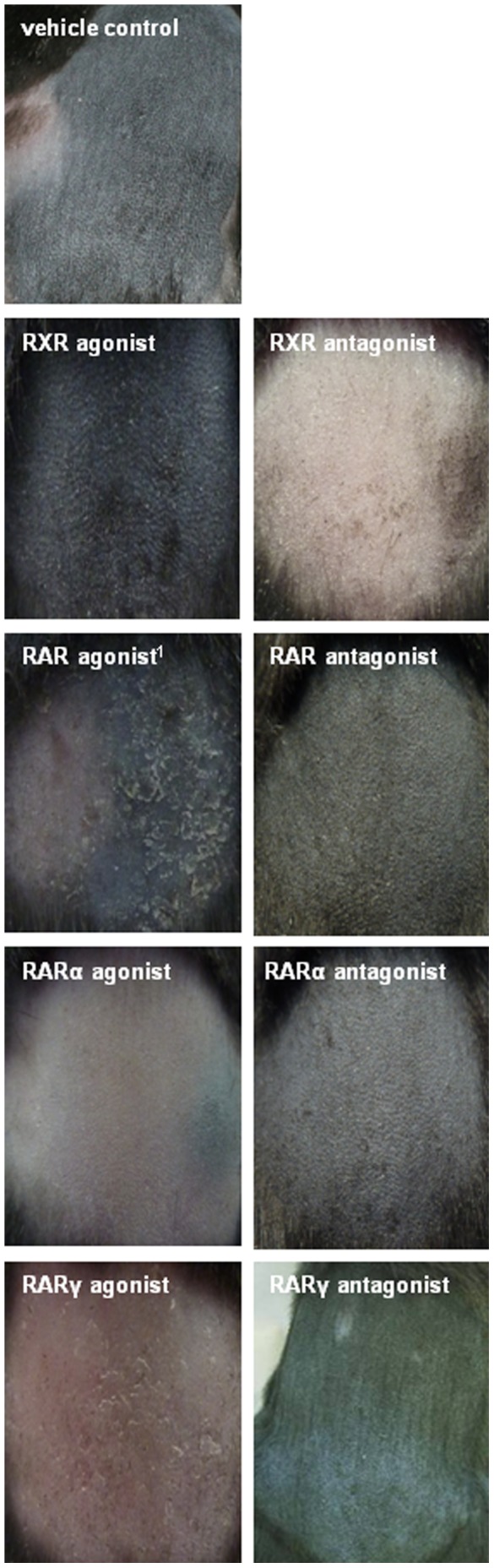
Back skin of mice after treatment with retinoid receptor-specific agonists and antagonists. Representative photographs of dorsal skin areas from mice topically treated with vehicle control (acetone), or various retinoid receptor-selective agonists or antagonists for 14 days. Note the scaly skin of mice treated with the synthetic RXR agonist or the synthetic RARγ agonist, and appearing most pronounced in the RAR agonist (ATRA) treated group. ^1^all-trans retinoic acid/ATRA.

**Figure 2:**
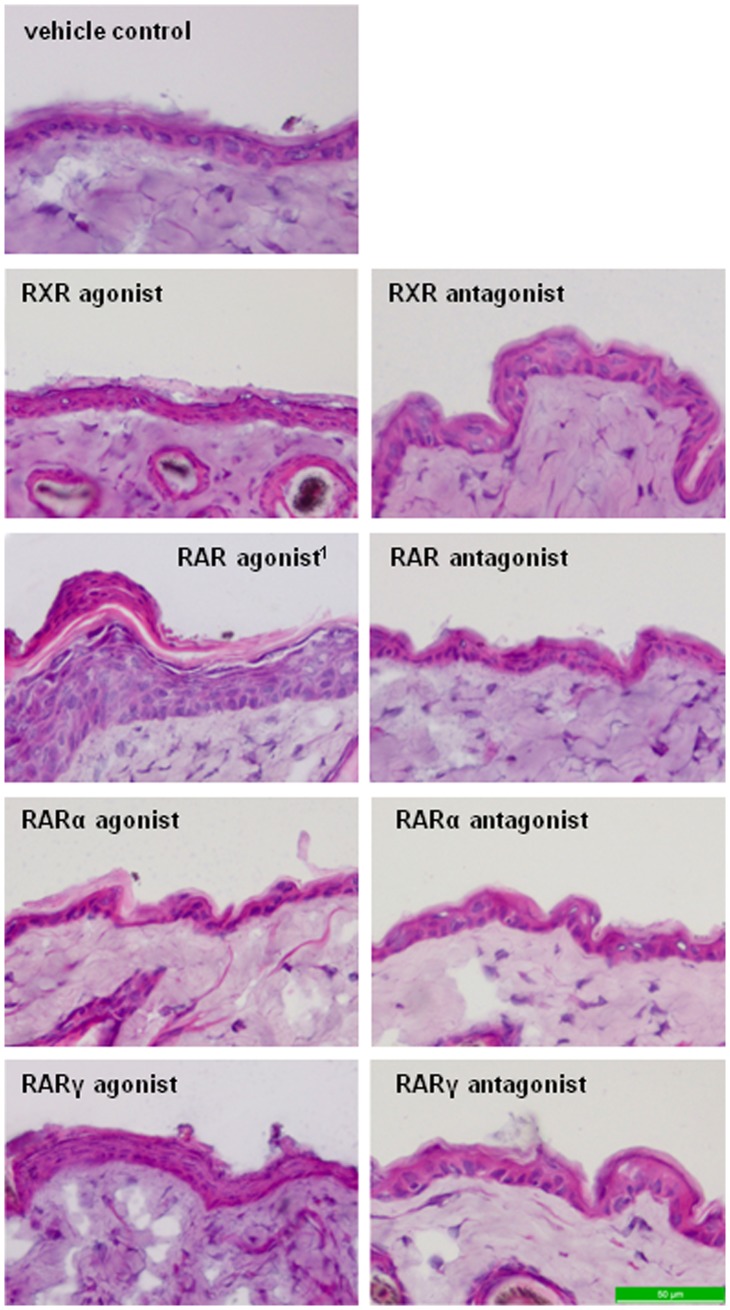
H+E stained skin sections of mice after treatment with retinoid receptor-specific agonists and antagonists. Representative photographs of H+E stained skin sections from mice topically treated with vehicle control (acetone), or various retinoid receptor-selective agonists or antagonists for 14 days. Note the epidermal thickness of mice treated with synthetic agonists for RXR or RARγ, and appearing most pronounced in the RAR agonist (ATRA) treated group. Epidermal thickness seemed comparable to vehicle control in mice treated with RARα agonist, RXR antagonist, RAR antagonist, and selective antagonists of RARα or RARγ. Original magnification (×20) was digitally magnified. ^1^all-trans retinoic acid/ATRA.

After two weeks topical treatment of mice with various retinoid receptor-specific agonists or antagonists, obvious signs of dryness (scales) could be observed in some groups compared to control mice. Representative images of the treated skin area at day 14 (end of treatment) are shown in Figure 1. Control animals were treated with acetone (vehicle) and their skin appeared normal without scales at the end of two weeks. Similar observations were made in the group treated with the RARα agonist showing only a very few scattered white scales on the back skin. In contrast, application of synthetic agonists for RXR or RARγ and the natural RAR ligand ATRA resulted in visibly dry and scaly skin. Compared to rather mild effects induced by the RXR agonist we could detect small scales already after the third treatment with the synthetic RARγ agonist. During the following days, number and size of scales increased and the skin appeared red and slightly shiny compared to control mice (Figure 1). Application of ATRA (same concentration as the synthetic RARγ agonist) showed the strongest effects resulting in apparently very dry skin with big white scales already shortly after initiating the treatment (not shown). Skin of these mice also seemed shiny compared to controls. Skin regions treated with receptor antagonists appeared mostly normal at day 14. A few small scales could be observed only after application of the RARα and RXR antagonists. In order to verify these visual impressions we also performed histological analysis (Figure 2). In accordance, epidermal thickness seemed comparable to control mice in all treatment groups except for mice treated with the synthetic RARγ agonist, RXR agonist or ATRA. Epidermal thickness was markedly increased in all three groups but appeared stronger in mice treated with the RARγ agonist and was most pronounced in ATRA-treated mice. Additionally, the epidermal surface seemed notably scaly after application of the synthetic RARγ agonist and ATRA (Figure 2).

### RAR-RXR Signaling Pathways Modify Epidermal Barrier Homeostasis

**Figure 3:**
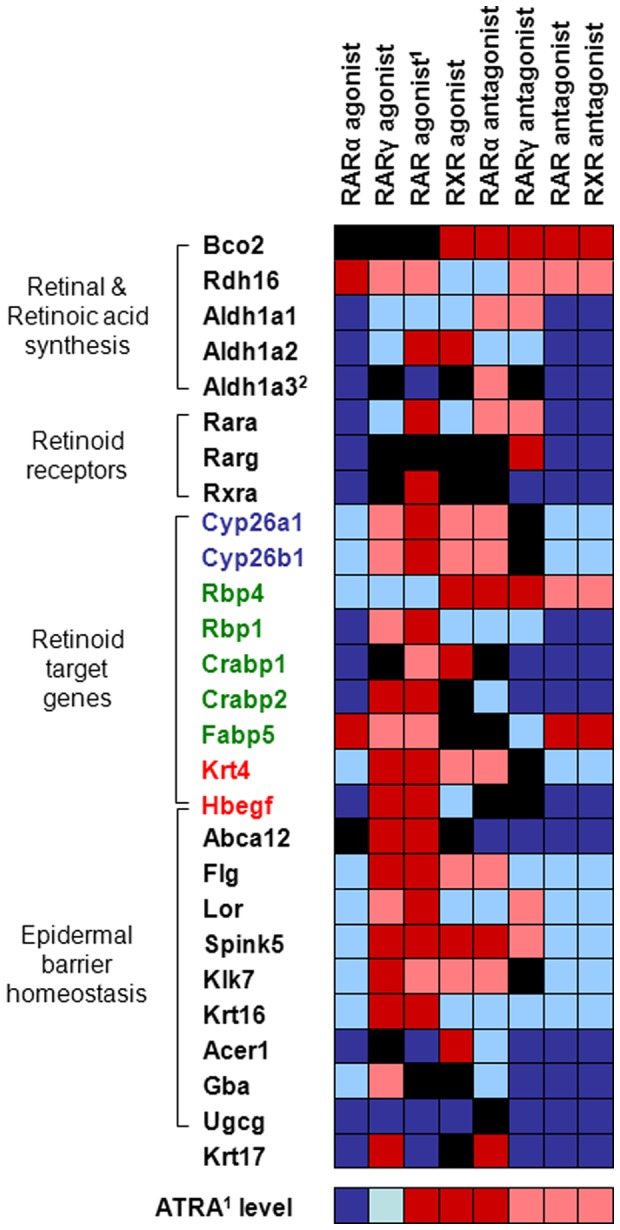
Altered gene expression after treatment with retinoid receptor-specific agonists and antagonists. Heat map displaying fold change of gene expression in mouse skin (n≥5/group) after treatment with retinoid receptor-specific agonists and antagonists compared to control mice (acetone). Genes are differentiated according to roles in retinoid metabolism or epidermal homeostasis. Retinoid target genes are further distinguished by specific function, i.e. retinoic acid synthesis (blue), retinoid transport (green), and genes unrelated to retinoid signaling (red). Color codes: dark red – significantly up-regulated; light red – non-significantly up-regulated; black – not regulated (±20%); light blue – non-significantly down-regulated; dark blue – significantly down-regulated. Statistical significance (*p*) is based on one-way ANOVA followed by Dunnett’s post test. A *p*-value <0.05 was considered significant. ^1^all-*trans* retinoic acid; ^2^also relevant as retinoid target gene.

We next investigated the expression of various genes with significant functions in epidermal barrier homeostasis upon treatment with receptor-specific agonists and antagonists. As shown in Table 1 and Figure 3, application of the synthetic RARγ agonist and ATRA both induced genes involved in barrier function (Abca12, Flg, Lor, Spink5, Krt16, Hbegf). On the other hand, mRNA levels of genes implicated in ceramide metabolism (Acer1, Gba, Ugcg) or cholesterol synthesis (Hmgcs2) were mainly decreased or unaffected by the treatment. Compared to RARγ ligand application, expression of these genes was markedly down-regulated (several times below detection limit) when mice were treated with the synthetic RARα agonist (Table 1, Figure 3). Noticeably, the same expression profile was observed after application of RAR or RXR antagonists. Treatment with the RXR agonist and RARα- and RARγ-specific antagonists resulted in inconsistent gene expressions with an increase of some genes (Spink5, Flg, Klk7) and decrease of other genes (Abca12, Krt16, Ugcg) involved in epidermal function (Table 1, Figure 3). Krt6b expression was below the limit of detection in all groups (not shown).

**Table 1:** Fold change of mRNA expression of genes involved in epidermal barrier homeostasis and chemotaxis in murine skin after two weeks of topical treatment with retinoid receptor-specific agonists or antagonists.

		Agonists (Fold change)				Antagonists (Fold change)			
Gene name	Symbol	RARα^1^	RARγ^2^	ATRA^3^	RXR^4^	RARα^5^	RARγ^6^	RAR^7^	RXR^8^
**Epidermal barrier homeostasis**									
ATP-binding cassette A12	Abca12	1±0.1	1.6±0.2^§^	1.5±0.1^§^	1±0.1	0.6±0.1*	0.5±0.1**	0.001±0.0003^§^	0.04±0.02^§^
Filaggrin	Flg	0.2±0.1	11.4±1.3^#^	32±4.4^§^	4.1±0.1	1.4±0.1	2.2±0.8	0.0003±0.0003	0.004±0.003
Involucrin	Ivl	0.9±0.1	1.6±0.1^§^	1.3±0.1	0.9±0.1	1±0.1	1.2±0.1	1.3±0.1	0.8±0.2
Loricrin	Lor	0.04±0.003	1.8±0.3	7.3±0.8^§^	0.6±0.1	0.7±0.2	1.3±0.3	0.002±0.0005	0.008±0.004
Transglutaminase 1	Tgm1	UDL^§^	0.7±0.2	2.5±0.2^§^	0.1±0.02^§^	0.4±0.1^#^	UDL^§^	UDL^§^	UDL^§^
Serine peptidase inhibitor,Kazal-type 5	Spink5	0.01±0.01	5.1±1^§^	2.8±0.4*	2.8±0.3^*^	5.1±0.6^§^	2.3±0.3	0.003±0.001	0.03±0.01
Kallikrein-related peptidase 5	Klk5	UDL	4.9±1.5^§^	2.8±0.4	1±0.1	2.4±0.5	3.4±0.7*	UDL	UDL
Kallikrein-related peptidase 7	Klk7	0.0008±0.0005	5.6±1.8^§^	2.7±0.3	3.2±0.6	2.1±0.3	1.2±0.1	0.005±0.001	0.02±0.008
Matrix metalloproteinase 9	Mmp9	0.4±0	2.7±0.5	2±1.1	0.8±0.1	4.7±0.8^§^	UDL	0.3±0.1	0.9±0.3
S100 calcium binding protein A7A	S100a7a	0.2±0.1	1.4±0.1	3.4±0.6^§^	0.8±0.1	0.02±0.002*	UDL^#^	UDL*	0.002±0.001*
Keratin 16	Krt16	0.02±0.004	3.8±0.4^§^	2.8±0.9*	0.6±0.1	0.5±0.1	UDL	0.03±0.006	0.03±0.01
Heparin-binding EGF-like growth factor^9^	Hbegf	0.03±0.01*	2.7±0.6^§^	2±0.2*	0.3±0.2	0.8±0.1	0.8±0.1	UDL*	UDL*
3-Hydroxy-3-methylglutaryl-CoA synthase 2	Hmgcs2	0.01±0.004^§^	0.5±0.1*	0.1±0.02^§^	1.3±0.3	0.6±0.1	UDL^§^	0.0006±0.0006^§^	0.1±0.02^§^
UDP-glucose ceramide glucosyltransferase	Ugcg	0.1±0.02^§^	0.4±0.1^§^	0.1±0.03^§^	0.2±0.02^§^	0.9±0.1	UDL^§^	0.002±0.002^§^	0.001±0.001^§^
Glucocerebrosidase	Gba	0.4±0.2	1.4±0.3	0.9±0.2	0.9±0.1	0.5±0.2	UDL^§^	UDL^§^	0.003±0.003^§^
Alkaline ceramidase 1	Acer1	UDL*	1.2±0.1	0.02±0.007^§^	1.5±0.2^*^	0.7±0.1	0.1±0.02^§^	UDL^§^	0.2±0.03^§^
**Immune response**									
Chemokine ligand 11/eotaxin-1	Ccl11	UDL^§^	0.6±0.1*	0.6±0.1*	0.5±0.1*	2±0.2^§^	UDL^§^	UDL^§^	UDL^§^
Chemokine ligand 24/eotaxin-2	Ccl24	UDL^§^	0.1±0.1^§^	0.009±0.009^§^	0.5±0.1*	1.9±0.2^§^	UDL^§^	UDL^§^	UDL^§^
Chemokine ligand 17/Tarc	Ccl17	2±0.6	2.7±0.4	9.6±1.5^§^	0.6±0.1	0.2±0.1	13.9±2.8^§^	UDL	0.1±0.1
Chemokine ligand 22/Mdc	Ccl22	UDL	4.3±1^§^	1.9±0.3	0.8±0.1	0.5±0.2	UDL	UDL	UDL
Keratin 17	Krt17	0.03±0.006^§^	1.5±0.1^§^	0.2±0.02^§^	1±0.1	1.3±0.1*	0.03±0.01^§^	0.04±0.004^§^	0.03±0.01^§^

1 BMS753; ^2^BMS961; ^3^all-*trans* retinoic acid; ^4^LG268; ^5^BMS614; ^6^UVI2041; ^7^BMS493; ^8^UVI3003; ^9^gene also relevant as retinoid target gene; UDL, under detection limit.

Fold change data are expressed as mean ± SEM (n≥5) and were determined in skin specimens of topically treated mice by qRT-PCR. Statistical significance (*p*) was tested using one-way ANOVA followed by Dunnett’s post test. *p<0.05, ^#^ *p*<0.01, ^§^ *p*<0.001, versus control (acetone).

### RAR-RXR Signaling Pathways Modify Skin-based Immune Responses

Retinoid-mediated signaling is known to play an important role in the immune system and a dysregulated retinoid metabolism was found in skin of atopic dermatitis patients (Mihály et al. 2011). Therefore, we investigated whether topical application of receptor-specific retinoids is sufficient to alter the expression of genes implicated in the immune response in skin, such as the chemokines Ccl11 (eotaxin-1), Ccl17 (Tarc), Ccl22 (Mdc), Ccl24 (eotaxin-2), Ccr3 and the inflammatory marker Krt17 (Table 1). The synthetic RXR activator exerted only a slight effect on gene expression in skin, while levels of chemokines and Krt17 were markedly decreased in response to the RARα agonist (except for Ccl17). Again, this result strongly resembled to those found after application of RAR or RXR antagonists. Topical treatment with the synthetic RARγ agonist and ATRA, as well as the RARγ antagonist decreased mRNA levels of Ccl11 and Ccl24 but induced Ccl17 and partly Ccl22, while it was the opposite in mouse skin treated with the RARα antagonist. Moreover, the chemokine receptor Ccr3 was below detection level regardless of which agonist or antagonist was applied (not shown). Expression of Krt17 was increased only in response to the RARγ agonist or RARα antagonist while it was decreased or unaltered in all other groups (Table 1).

### RARα and RARγ Differentially Regulate Retinoid-mediated Signaling

Moreover, we were interested in the effect of RAR subtype-selective agonists on retinoid metabolism. Interestingly, we found that treatment with the synthetic RARα agonist down-regulated the expression of all investigated genes with a role in retinoid metabolism that is RA synthesis, retinoid receptors and target genes (Table 2, Figure 3). Only mRNA levels of the lipid transporter Fabp5 and an enzyme involved in retinal synthesis (Rdh16) were significantly increased by the agonist. In contrast, the synthetic agonist for RARγ and ATRA, which is a natural RAR agonist, induced the expression of nearly all retinoid target genes in the skin of mice, e.g. Cyp26a1, Cyp26b1, Rbp1, Crabp1, Hbegf and Krt4 as a marker for retinoid activity (Table 2, Figure 3). Similarly, topical application of the RXR-selective agonist induced the expression of some retinoid target genes (Cyp26a1, Cyp26b1, Rbp4, Crabp1, Krt4), but the treatment did not affect or slightly decrease the expression of other targets (Crabp2, Fabp5, Rbp1, Hbegf). Repetitive treatment with the RARγ-selective agonist showed no significant effect on retinal and RA-synthesis enzymes, and retinoid receptor gene expression in skin. However, the endogenous RAR ligand ATRA and the RXR agonist markedly increased mRNA levels of Aldh1a2 and ATRA further induced Rara and Rxra gene expression, while it decreased Aldh1a3 expression in skin (Table 2, Figure 3).

**Table 2:** Fold change of retinoid metabolism-related gene expression in skin of mice after two weeks topical treatment with retinoid receptor-specific agonists.

		Agonists (Fold change)			
Gene name	Symbol	RARα^1^	RARγ^2^	ATRA^3^	RXR^4^
**Retinal synthesis**					
Beta-carotene oxygenase	Bco2	UDL	UDL	UDL	3305±192^§^
Short chain dehydrogenase/reductase 16C5	Sdr16c5	UDL	0.6±0.1	9.4±1.3^§^	0.9±0.1
Retinol dehydrogenase 10	Rdh10	0.01±0.003^§^	1.5±0.1^§^	0.7±0.1*	0.6±0.1^#^
Retinol dehydrogenase 16	Rdh16	493±128^§^	1.3±0.3	2.5±0.3	0.5±0.1
Alcohol dehydrogenase 7	Adh7	0.2±0.1	1.8±0.3	2.8±0.5^§^	2±0.1^*^
**Retinoic acid synthesis**					
Aldehyde dehydrogenase 1A1	Aldh1a1	UDL^#^	0.7±0.1	0.6±0.1	0.3±0.1
Aldehyde dehydrogenase 1A2	Aldh1a2	0.04±0.009*	0.7±0.2	3.4±0.5^§^	2.4±0.3^#^
Aldehyde dehydrogenase 1A3^5^	Aldh1a3	UDL^§^	1±0.04	0.02±0.008^§^	0.9±0.2
**Retinoid receptor**					
Retinoic acid receptor α	Rara	UDL*	0.3±0.1	5.8±0.4^§^	0.7±0.1
Retinoic acid receptor β^5^	Rarb	UDL^§^	1.1±0.2	1.4±0.1*	0.6±0.1*
Retinoic acid receptor γ	Rarg	UDL^§^	1.1±0.1	0.9±0.3	0.9±0.1
Retinoid X receptor α	Rxra	0.002±0.0006^§^	0.9±0.1	1.4±0.1^§^	1±0.1
**Retinoid target genes**					
*Retinoic acid degradation*					
Cytochrome P450 26A1	Cyp26a1	UDL	19±5.4	1410±161^§^	1.5±0.3
Cytochrome P450 26B1	Cyp26b1	0.003±0.003	13±0.5	299±54^§^	1.6±0.2
Cytochrome P450 2S1	Cyp2s1	UDL^#^	0.9±0.1	0.6±0.1	0.9±0.1
*Retinoid transport proteins*					
Retinol binding protein 4	Rbp4	UDL	UDL	UDL	2816±244^§^
Cellular retinol binding protein 1	Rbp1	0.02±0.004^§^	1.4±0.2	2.8±0.2^§^	0.7±0.1
Cellular retinoic acid binding protein 1	Crabp1	0.02±0.01^#^	1±0.2	1.7±0.3	1.8±0.2*
Cellular retinoic acid binding protein 2	Crabp2	0.0007±0.0003^§^	1.5±0.2*	2±0.2^§^	0.8±0.04
Fatty acid binding protein 5	Fabp5	10±0.8^§^	1.9±0.4	2.2±0.1	1.2±0.1
*Retinol esterification*					
Lecithin-retinol acyltransferase	Lrat	0.02±0.007^§^	2.3±0.2^§^	2±0.1^§^	1±0.1
Diacylglycerol O-acyltransferase	Dgat	UDL^§^	1.5±0.2^§^	0.4±0.1^§^	0.2±0.03^§^
*Further retinoid target genes^6^*					
Keratin 4	Krt4	UDL	6470±646^§^	3167±679^§^	7.7±3
Retinoic acid receptor responder 2	Rarres2	UDL	1.8±0.2	1.3±0.3	1.6±0.2
Heparin-binding EGF-like growth factor^7^	Hbegf	0.03±0.01*	2.7±0.6^§^	2±0.2*	0.3±0.2

1 BMS753; ^2^BMS961; ^3^all-*trans* retinoic acid; ^4^LG268; ^5^retinoid target genes; ^6^target genes not involved in retinoid signaling; ^7^gene also relevant in epidermal homeostasis; UDL, under detection limit. Fold change data are expressed as mean ± SEM (n≥5) and were determined in skin specimens of topically treated mice by qRT-PCR. Statistical significance (*p*) was tested using one-way ANOVA followed by Dunnett’s post-test. *p<0.05, ^#^ *p*<0.01, ^§^ *p*<0.001, versus control (acetone).

### RAR and RXR Antagonists Decrease the Expression of Genes Involved in Retinoid Signaling in Skin

Topical application of antagonists for RARα and RARγ resulted in non-significantly reduced or unaltered expression of several genes involved in retinoid signaling in skin. However, some genes seemed to be slightly induced by both antagonists, such as Bco2, Rbp4, Aldh1a1 which is responsible for RA synthesis, Rara, Rarg and some target genes like Cyp26a1, Cyp26b1 and Krt4 (Figure 3, Table 3). In contrast, antagonists for RAR and RXR decreased the expression of nearly all of these genes below detection limit. Only mRNA levels of Bco2, Rdh16, Rbp4 and Fabp5 were found to be elevated by the antagonists. Surprisingly, this expression pattern strongly resembled to that which we observed in skin of mice treated with the synthetic RARα agonist (Figure 3, Table 3).

**Table 3:** Fold change of retinoid metabolism-related gene expression in skin of mice after two weeks topical treatment with retinoid receptor-specific antagonists.

		Antagonists (Fold change)			
Gene name	Symbol	RARα^1^	RARγ^2^	RAR^3^	RXR^4^
**Retinal synthesis**					
Beta-carotene oxygenase	Bco2	443±84^§^	124±78	90±41	117±14
Short chain dehydrogenase/reductase 16C5	Sdr16c5	0.7±0.04	0.3±0.04	UDL	UDL
Retinol dehydrogenase 10	Rdh10	0.8±0.1	0.1±0.03^§^	0.003±0.003^§^	0.1±0.04^§^
Retinol dehydrogenase 16	Rdh16	0.02±0.01	3.5±0.7	6.5±1.2	1.3±0.5
Alcohol dehydrogenase 7	Adh7	0.01±0.001*	1±0.1	0.002±0.0004^#^	0.006±0.002*
**Retinoic acid synthesis**					
Aldehyde dehydrogenase 1A1	Aldh1a1	1.3±0.3	1.5±0.3	UDL^#^	UDL^#^
Aldehyde dehydrogenase 1A2	Aldh1a2	0.5±0.1	0.2±0.03	0.1±0.1*	0.03±0.02*
Aldehyde dehydrogenase 1A3^5^	Aldh1a3	1.3±0.2	1±0.2	0.003±0.001^§^	0.004±0.003^§^
**Retinoid receptor**					
Retinoic acid receptor α	Rara	1.5±0.3	1.3±0.1	UDL*	UDL*
Retinoic acid receptor β^5^	Rarb	0.6±0.03	0.7±0.1	UDL^§^	UDL^§^
Retinoic acid receptor γ	Rarg	0.8±0.1	1.7±0.2^#^	UDL^§^	UDL^§^
Retinoid X receptor α	Rxra	0.9±0.1	0.8±0.1*	0.0004±0.0002^§^	0.01±0.005^§^
**Retinoid target genes**					
*Retinoic acid degradation*					
Cytochrome P450 26A1	Cyp26a1	1.6±0.6	0.9±0.3	UDL	UDL
Cytochrome P450 26B1	Cyp26b1	1.3±0.2	1.1±0.2	0.001±0.001	0.02±0.009
Cytochrome P450 2S1	Cyp2s1	0.9±0.1	1.6±0.1*	UDL^§^	0.006±0.004^#^
*Retinoid transport proteins*					
Retinol binding protein 4	Rbp4	448±18^§^	1710±505^§^	12±12	30±30
Cellular retinol binding protein 1	Rbp1	0.6±0.1	0.7±0.04	0.003±0.002^§^	0.02±0.003^§^
Cellular retinoic acid binding protein 1	Crabp1	1.1±0.3	UDL^§^	0.02±0.01^#^	0.1±0.03^#^
Cellular retinoic acid binding protein 2	Crabp2	0.7±0.1	0.5±0.04^#^	UDL^§^	0.04±0.01^§^
Fatty acid binding protein 5	Fabp5	0.9±0.1	0.2±0.02	13±0.6^§^	8±1^§^
*Retinol esterification*					
Lecithin-retinol acyltransferase	Lrat	1.2±0.1	0.6±0.1	0.003±0.002^§^	0.01±0.007^§^
Diacylglycerol O-acyltransferase	Dgat	0.2±0.02^§^	0.1±0.008^§^	UDL^§^	0.003±0.003^§^
*Further retinoid target genes^6^*					
Keratin 4	Krt4	154±37	UDL	UDL	UDL
Retinoic acid receptor responder 2	Rarres2	3.4±0.6^§^	1±0.3	UDL	0.005±0.005
Heparin-binding EGF-like growth factor^7^	Hbegf	0.8±0.1	0.8±0.1	UDL	UDL*

1 BMS614; ^2^UVI2041; ^3^BMS493; ^4^UVI3003; ^5^retinoid target genes; ^6^target genes not involved in retinoid signaling; ^7^gene also relevant in epidermal homeostasis; UDL, under detection limit. Fold change data are expressed as mean ± SEM (n≥5) and were determined in skin specimens of topically treated mice by qRT-PCR. Statistical significance (*p*) was tested using one-way ANOVA followed by Dunnett’s post-test. *p<0.05, ^#^ *p*<0.01, ^§^ *p*<0.001, versus control (acetone).

### RXR Agonist and RARα Antagonist Increase ATRA Levels in Skin via Induced Synthesis

ATRA levels in skin were found to be differentially affected depending on the applied receptor-selective agonist or antagonist (Figure 3, Table S1). Concentrations of ATRA were significantly decreased in the skin of mice treated with the synthetic RARα agonist and non-significantly by the RARγ agonist. Furthermore, treatments with antagonists for RARγ, RARs, or RXRs resulted in elevated ATRA concentrations, while only RARα antagonist treatment induced a significant increase. As expected, we found ATRA levels markedly elevated upon treatment with this RAR agonist itself (highest level among all groups). Noticeably, however, was the pronounced elevation of ATRA in mouse skin after application of the synthetic RXR agonist (Figure 3, Table S1).

## Discussion

In the present study we repetitively treated mice topically with various retinoid receptor-specific agonists or antagonists in order to determine the effect of selective retinoid-mediated signaling in skin on epidermal barrier homeostasis, immune regulation and retinoid metabolism. The main finding of this study was the strong difference between the positive retinoid-mediated signaling via RARγ pathways in contrast to the negative retinoid-mediated signaling via RARα in the skin.

Epidermal hyperproliferation is a well established effect of RAR-activation in skin [2], [37], [38] and was induced in this study by ATRA and the synthetic RARγ agonist (Figure 1 and 2), which was further supported by an induced expression of regulators of desquamation such as Spink5, Klk5 and Klk7 [39]–[41]. Moreover, elevated mRNA levels of Hbegf and Krt16, which were already related previously with induced keratinocyte proliferation [2], [42]–[44], also contributed to the result (Table 1, Figure 3). Somewhat surprising, however, was the mild induction of epidermal proliferation by the synthetic RXR agonist since no such observation was reported in a previous study using another synthetic RXR agonist [2]. Retinoid effects in skin are most likely mediated by RARγ-RXR heterodimers while their transcriptional activity is dependent on the RAR-activating ligand [2], [3]. Upon treatment with the RXR agonist we observed increased Aldh1a2 gene expression and elevated ATRA levels in skin (Table 2 and S1), indicating induced ATRA synthesis which might account for the mild epidermal hyperproliferation, most probably mediated by the RAR partner. However, another RXR heterodimer partner, PPARδ, was previously found to be implicated in the regulation of keratinocyte hyperproliferation [45]–[47]. Compared to RAR-RXR, this heterodimer is permissive which means an RXR ligand is sufficient to activate transcription of respective target genes [48]. This might suggest alternative pathways to be involved in RXR agonist-induced hyperproliferation.

Moreover, retinoid application affected various other processes in skin, as indicated by altered expression levels of genes involved in epidermal barrier homeostasis such as Abca12, Flg, and Lor [49], [50] and of genes with roles in lipid barrier formation and ceramide metabolism, e.g. Hmgcs2, Ugcg, Gba, Acer1 [51]–[54]. Consistently, such retinoid-mediated effects have already been reported by Lee at al. (2009) in epidermal keratinocytes [42].

These results strongly suggest that retinoid-mediated signaling is required for normal barrier homeostasis and that retinoid-induced dysregulation may be a predisposing factor for dermatological diseases. Thereby both, antagonism and induction of RAR- and/or RXR-mediated signaling in skin appear to be able to disturb barrier homeostasis as shown in our study and previous works [55]–[59]. However, no further functional analysis, such as determination of trans-epidermal water loss, was performed in order to prove barrier disturbance.

It is well established that retinoids play important roles in the immune system [16], [60], especially in Th2-type cell differentiation [61]–[63]. Interestingly, the expression of various chemokines which are preferentially attracting Th2-type lymphocytes during inflammatory processes [64]–[66] was differently altered by the retinoids applied in the present study (Table 1). However, undetectable mRNA levels of the corresponding chemokine receptor (CCR3) which is expressed by infiltrating immune competent cells such as eosinophils [67]–[69] might suggest the absence of inflammatory cells in the skin upon retinoid treatments. These results indicate that topical retinoids can modify potential immune responses by altering chemokine expression of resident skin cells and that the outcome of immune alterations seems to differ depending on the RAR subtype activated.

Retinoid receptor agonist treatment affected the expression of all genes investigated in the skin and involved in retinoid-mediated signaling (retinoid metabolism, transport, target genes) in general oppositely to antagonists. Likewise, target genes were mainly induced after treatment with ATRA or the RARγ agonist (Figure 3 and Table 2), as previously reported [38], [42], [70], [71]. Moreover, both agonists induced very similar gene expression patterns and given the fact that RARγ is the predominant RAR subtype in skin [5] it is indicated that ATRA mediates its activity in skin through RARγ rather than RARα [2], [5], [72]. Most interesting, however, was a consequent down-regulation of gene expression by the synthetic RARα agonist which is in line with reduced ATRA levels in mouse skin, possibly due to decreased ATRA synthesis via Aldh enzymes (Table 2). Only Fabp5 and Rdh16 expressions were increased in response to the agonist. This expression pattern strongly resembled to that in response to RAR or RXR antagonists while both antagonists further seemed to induce Bco2 and Rbp4 expression (Figure 3, Tables 2 and 3). The proteins encoded by those genes are implicated in retinoid metabolism and transport [73]–[76]. Thus, it seems plausible that ATRA or retinoid derivatives different from ATRA, like oxo-retinoids or still unknown endogenous RAR ligands could be generated upon retinoid receptor antagonism and shuttled to nuclear receptors different from RARs, as it was already proposed for Fabp5-mediated ATRA-induced PPARδ activation [73], [77], [78]. Additionally, also NR4A1/NUR77 and RXR were shown to form heterodimers which respond to RXR activators *in vivo* and *in vitro* [79] and might thereby participate in retinoid-mediated signaling when RARs are antagonized. Moreover, Volakakis et al. [80] demonstrated that NR4A1/NUR77 can induce the expression of Fabp5 in HEK293 cells which potentially enhances RA-mediated PPARδ signaling. Interestingly, we found Nr4a1/Nur77 and Ppard expression in skin significantly decreased or below detection limit in response to those ligands which markedly induced Fabp5 expression, namely the RARα agonist, RAR and RXR antagonists (Tables 2 and 3, Table S2). This may be indicative of (late) negative feedback regulations on the gene expression level in response to induced Fabp5 expression. Whether FABP5-mediated PPARδ signaling and/or a novel, as yet undetermined retinoid(s) might mediate such an alternative retinoid pathway in skin is currently under investigation in our laboratory. Moreover, since mRNA levels of ATRA-synthesizing enzymes (Aldhs) following RAR and RXR antagonist application were not in accord with elevated ATRA levels in the skin of those mice, we suggest that ATRA synthesis upon antagonist treatment may be mediated by Bco2, Rdh16, RBP4 and/or other pathways, from precursors present in the skin and/or via transporter-mediated pathways delivering retinoids to the skin [74].

**Figure 4:**
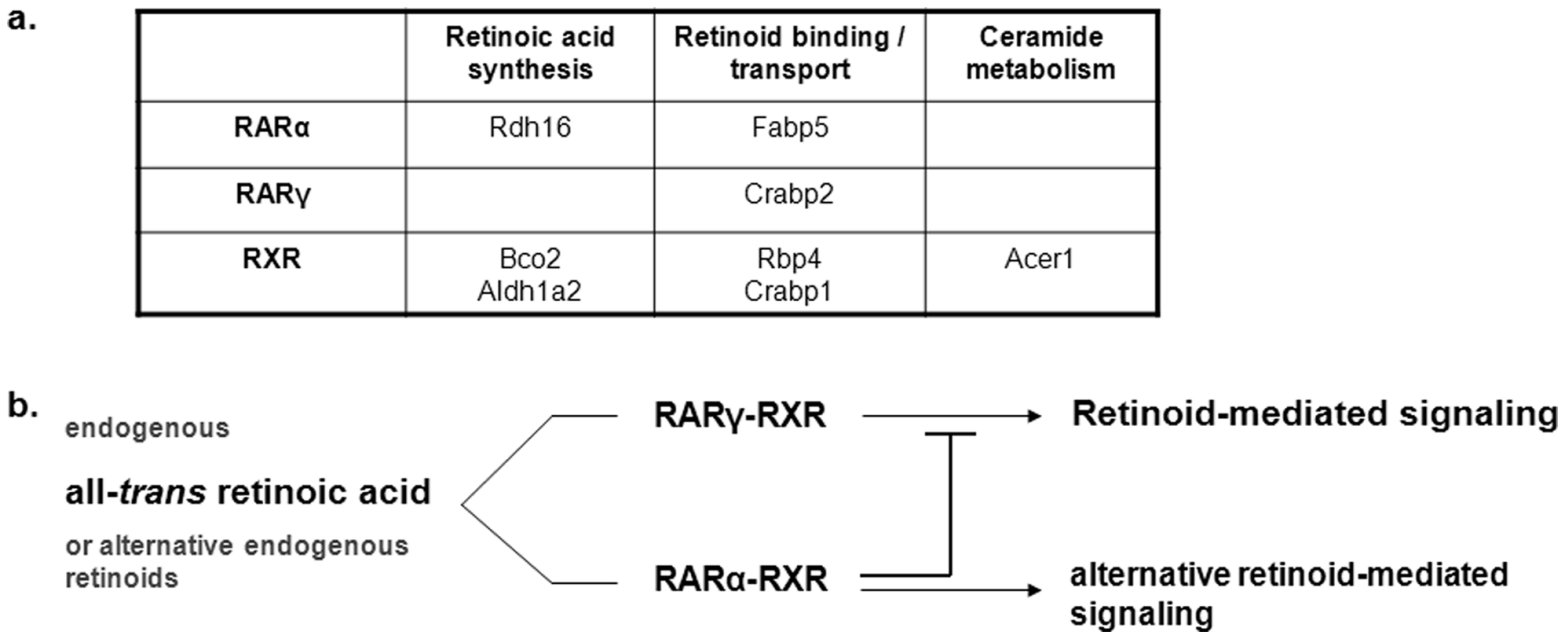
Retinoid receptor-selective gene regulation. (**a**) Retinoid receptor-selective induction of genes with specific roles in retinoid signaling or epidermal barrier homeostasis in skin of mice treated topically with selective agonists for RARα, RARγ or RXR for 14 days. (**b**) Proposed outcome of selective signaling via RARα-RXR or RARγ-RXR in skin of mice induced by endogenous retinoids, such as all-*trans* retinoic acid.

Altogether, our observations indicate different roles of RXR-, RARα- and RARγ-mediated signaling pathways in skin (Figure 4a) and suggest that induction of RARα signaling might result in the suppression of RARγ-mediated pathways in the skin of mice. Considering the induced RARα gene expression after topical ATRA treatment, this appears to be an efficient physiological switch to different retinoid-mediated signaling pathways. However, it is unknow how RARα mediates its suppressive action on RARγ signaling. High RARα expression was found in inflammatory cells infiltrating the skin in several dermatoses [81], however, in normal skin its expression level is fairly low compared to RARγ molecules [5]. Thus it seems unlikely that a competition between both receptors for RXRα as heterodimer partner could be the explanation. Instead, RARα apparently regulates the expression of different sets of genes, possibly also in different skin cell types, than does RARγ and might also induce the transcription of co-repressor molecules upon activation.

In summary, this study lets us emphasize that there must be yet unidentified alternative retinoid signaling pathways or a broader range of endogenous retinoids present in skin for selective RARα, RARγ, or RXR activation as outlined in Figure 4b. Moreover, our data indicate that unbalanced retinoid signaling in the skin mediated by RARα, RARγ and/or RXR signaling pathways as well as potential unidentified pathways, affects epidermal barrier homeostasis and skin-based immune responses in mice. This retinoid dysregulation may play a central role in various skin diseases and the obtained data from this study might help to identify appropriate treatment strategies for diseased skin with dysregulated retinoid signaling using selective RAR and RXR agonists or antagonists, alone or in combination.

## Supporting Information

Figure S1 **Synthesis of BMS753.** Reagents and conditions: a. AlCl_3_, C_6_H_6_, 100°C, 4 h (65%). b. KMnO_4_, H_2_O, NaOH, 100°C, 3 h (78%). c. CrO_3_, AcOH, 25°C, 4 h (93%). d. AlCl_3_, ClCOCO_2_Et, CH_2_Cl_2_, 25°C, 2 h (43%). e. NaOH (1 N, aq), MeOH, 25°C, 1 h (99%). f. NaOH, MeOH, H_2_O_2_, 25°C, 16 h (96%). g. i) Oxalyl chloride, CH_2_Cl_2_, DMF, 5 min. ii) Methyl 4-aminobenzoate, pyridine, 25°C, 16 h (45%). h. NaOH (1 N, aq), MeOH, 70°C, 4 h (89%).
(TIF)

Figure S2 **Synthesis of BMS189961.** Reagents and conditions: a. i) *t*-Butyllithium, THF, −78°C, 30 min. ii) (COCO_2_Me)_2_, THF, 25°C, 16 h (88%) b. LiOH·H_2_O, 4 h, 25°C (76%) c. i) Oxalyl chloride, DMF. ii) Ethyl 4-amino-3-fluorobenzoate, Et_3_N, EtOAc, 16 h, 25°C (65%). d. NaBH_4_, MeOH, 5 min, (79%) e. LiOH·H_2_O, 25°C, 4 h (64%).
(TIF)

Figure S3 **Synthesis of BMS614.** Reagents and conditions: a) HBF_4_, NaNO_2_, H_2_O, 10°C, 89%. b) H_2_SO_4_, H_2_O, reflux, 1 h, 88%. c) Tf_2_O, Py, 25°C, 16 h, 100%. d) Pd(OAc)_2_, dppp, CO, Et_3_N, MeOH, DMSO, 70°C, 3 h, 93%. e) *i.* 3-bromoquinoline, *n*-BuLi, THF, −78°C. *ii.* THF, 25°C, 2 h, 32%. f) *p*-TsOH, toluene, 90°C, 2.5 h, 83%. g) NaOH (10 M), EtOH/H_2_O (1∶1), 25°C, 24 h, 88%. h) *i.* (ClCO)_2_, CH_2_Cl_2_, DMF, 25°C, 2 h; *ii.* methyl 4-aminobenzoate, Py, 25°C, 2 h, 26%. i) NaOH (10 M), EtOH/H_2_O (1∶1), 25°C, 24 h, 28%.
(TIF)

Figure S4 **Synthesis of UVI2041.** Reagents and conditions: a) EDC (1.1 equiv), DMAP (0.01 equiv), Trimethylsilylethanol (1.1 equiv), CH_2_Cl_2,_ 18 h, 23°C, 65%. b) NH_2_OH (2 equiv), pyridine (2.2 equiv), EtOH, 70°C, 20 h, 66% (*E/Z* isomer mixture at the oxime). c) TBAF (2 equiv), DMSO, 30 min, 63%.
(TIF)

Table S1 ATRA concentrations (ng/g) in murine skin after two weeks topical treatment with retinoid receptor-selective agonists or antagonists.
(DOCX)

Table S2 Fold change of mRNA expression of Nr4a1 and Ppard in skin of mice after two weeks of topical treatment with retinoid receptor-specific agonists or antagonists.
(DOCX)
