# Supplementary figures and images for: Regulation of Retinoid-Mediated Signaling Involved in Skin Homeostasis by RAR and RXR Agonists/Antagonists in Mouse Skin
Source: PLoS One. 2013 Apr 24;8(4):e62643. doi: 10.1371/journal.pone.0062643 (PMC3634743; doi:10.1371/journal.pone.0062643)

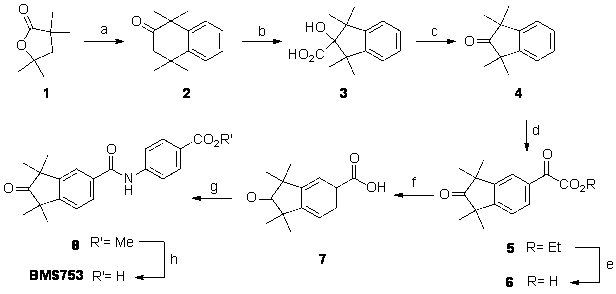

Supplement: Figure S1 — Synthesis of BMS753. Reagents and conditions: a. AlCl3, C6H6, 100°C, 4 h (65%). b. KMnO4, H2O, NaOH, 100°C, 3 h (78%). c. CrO3, AcOH, 25°C, 4 h (93%). d. AlCl3, ClCOCO2Et, CH2Cl2, 25°C, 2 h (43%). e. NaOH (1 N, aq), MeOH, 25°C, 1 h (99%). f. NaOH, MeOH, H2O2, 25°C, 16 h (96%). g. i) Oxalyl chloride, CH2Cl2, DMF, 5 min. ii) Methyl 4-aminobenzoate, pyridine, 25°C, 16 h (45%). h. NaOH (1 N, aq), MeOH, 70°C, 4 h (89%). (TIF) [file pone.0062643.s001.tif]

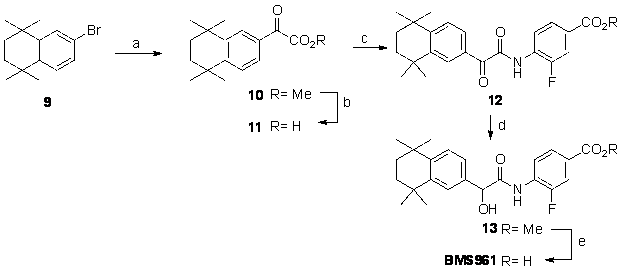

Supplement: Figure S2 — Synthesis of BMS189961. Reagents and conditions: a. i) t-Butyllithium, THF, −78°C, 30 min. ii) (COCO2Me)2, THF, 25°C, 16 h (88%) b. LiOH·H2O, 4 h, 25°C (76%) c. i) Oxalyl chloride, DMF. ii) Ethyl 4-amino-3-fluorobenzoate, Et3N, EtOAc, 16 h, 25°C (65%). d. NaBH4, MeOH, 5 min, (79%) e. LiOH·H2O, 25°C, 4 h (64%). (TIF) [file pone.0062643.s002.tif]

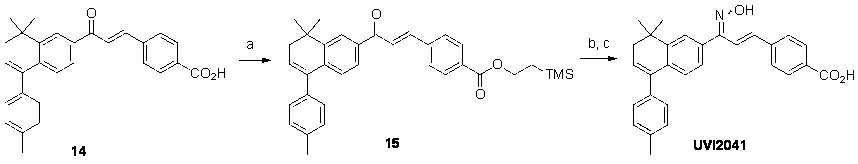

Supplement: Figure S4 — Synthesis of UVI2041. Reagents and conditions: a) EDC (1.1 equiv), DMAP (0.01 equiv), Trimethylsilylethanol (1.1 equiv), CH2Cl2, 18 h, 23°C, 65%. b) NH2OH (2 equiv), pyridine (2.2 equiv), EtOH, 70°C, 20 h, 66% (E/Z isomer mixture at the oxime). c) TBAF (2 equiv), DMSO, 30 min, 63%. (TIF) [file pone.0062643.s004.tif]
