# Supplementary material for: Regulation of Retinoid-Mediated Signaling Involved in Skin Homeostasis by RAR and RXR Agonists/Antagonists in Mouse Skin
Source: PLoS One. 2013 Apr 24;8(4):e62643. doi: 10.1371/journal.pone.0062643 (PMC3634743; doi:10.1371/journal.pone.0062643)
Supplement: Table S1 — ATRA concentrations (ng/g) in murine skin after two weeks topical treatment with retinoid receptor-selective agonists or antagonists. (DOCX) [file pone.0062643.s005.docx]

**Table S1. ATRA concentration (ng/g) in murine skin after two weeks topical treatment with retinoid receptor-selective agonists or antagonists.**

|  |  | **Agonists** | | | | **Antagonists** | | | |
| --- | --- | --- | --- | --- | --- | --- | --- | --- | --- |
|  | **acetone** | **RARα^1^** | **RARγ^2^** | **ATRA^3^** | **RXR^4^** | **RARα^5^** | **RARγ^6^** | **RAR^7^** | **RXR^8^** |
| All-*trans* retinoic acid | 2.5 ± 0.4 | 1.1 ± 0.4^*^ | 1.8 ± 1.1 | 5001 ± 1127^#^ | 25 ± 9.4^*^ | 14 ± 4.2^*^ | 4.3 ± 1.7 | 5.6 ± 1.6 | 4.6 ± 2 |

^1^ BMS753; ^2^ BMS961; ^3^ all-*trans* retinoic acid; ^4^ LG268; ^5^ BMS614; ^6^ UVI2041; ^7^ BMS493; ^8^ UVI3003

Concentrations were determined in skin specimens of topically treated mice (n≥5) by HPLC MS-MS and were calculated as ng/g skin. Data are indicated as mean ± SEM.

Statistical significance (*p*) was tested using Student’s *t*-test. **p*<0.05, #*p*<0.005, versus control (acetone)
