# Supplementary material for: Regulation of Retinoid-Mediated Signaling Involved in Skin Homeostasis by RAR and RXR Agonists/Antagonists in Mouse Skin
Source: PLoS One. 2013 Apr 24;8(4):e62643. doi: 10.1371/journal.pone.0062643 (PMC3634743; doi:10.1371/journal.pone.0062643)
Supplement: Table S2 — Fold change of mRNA expression of Nr4a1 and Ppard in skin of mice after two weeks of topical treatment with retinoid receptor-specific agonists or antagonists. (DOCX) [file pone.0062643.s006.docx]

**Table S2. Fold change of mRNA expression of Nr4a1 and Ppard in skin of mice after two weeks of topical treatment with retinoid receptor-specific agonists or antagonists.**

|  |  | **Agonists** (Fold change) | | | | **Antagonists** (Fold change) | | | |
| --- | --- | --- | --- | --- | --- | --- | --- | --- | --- |
| **Gene name** | **Symbol** | **RARα^1^** | **RARγ^2^** | **ATRA^3^** | **RXR^4^** | **RARα^5^** | **RARγ^6^** | **RAR^7^** | **RXR^8^** |
| Nuclear receptor family 4, group A, number 1 | Nr4a1 / Nurr77 | 0.1 ± 0.04^#^ | 0.9 ± 0.1 | 0.7 ± 0.1 | 0.4 ± 0.04 | 0.007 ± 0.001^#^ | 1.2 ± 0.1 | UDL | UDL |
| Peroxisome proliferator-activated receptor delta | Ppard | 0.03 ± 0.01^#^ | 1.1 ± 0.1 | 0.9 ± 0.1 | 0.8 ± 0.04^*^ | 1 ± 0.1 | UDL | 0.0009 ± 0.0009^#^ | 0.04 ± 0.01^#^ |

^1^ BMS753; ^2^ BMS961; ^3^ all-*trans* retinoic acid; ^4^ LG268; ^5^ BMS614; ^6^ UVI2041; ^7^ BMS493; ^8^ UVI3003

UDL, under detection limit

Fold change data are expressed as mean ± SEM (n≥5) and were determined in skin specimesn of topically treated mice by qRT-PCR. Statistical significance (*p*) was tested using one-way ANOVA followed by Dunnett’s post-test. **p*<0.05, #p<0.001, versus control (acetone)
